# Supplementary material for: Spin-liquid-like state in a spin-1/2 square-lattice antiferromagnet perovskite induced by d10–d0 cation mixing
Source: Nat Commun. 2018 Mar 14;9:1085. doi: 10.1038/s41467-018-03435-1 (PMC5852160; doi:10.1038/s41467-018-03435-1)
Supplement: Supplementary file 1 — Supplementary Information [file 41467_2018_3435_MOESM1_ESM.pdf]

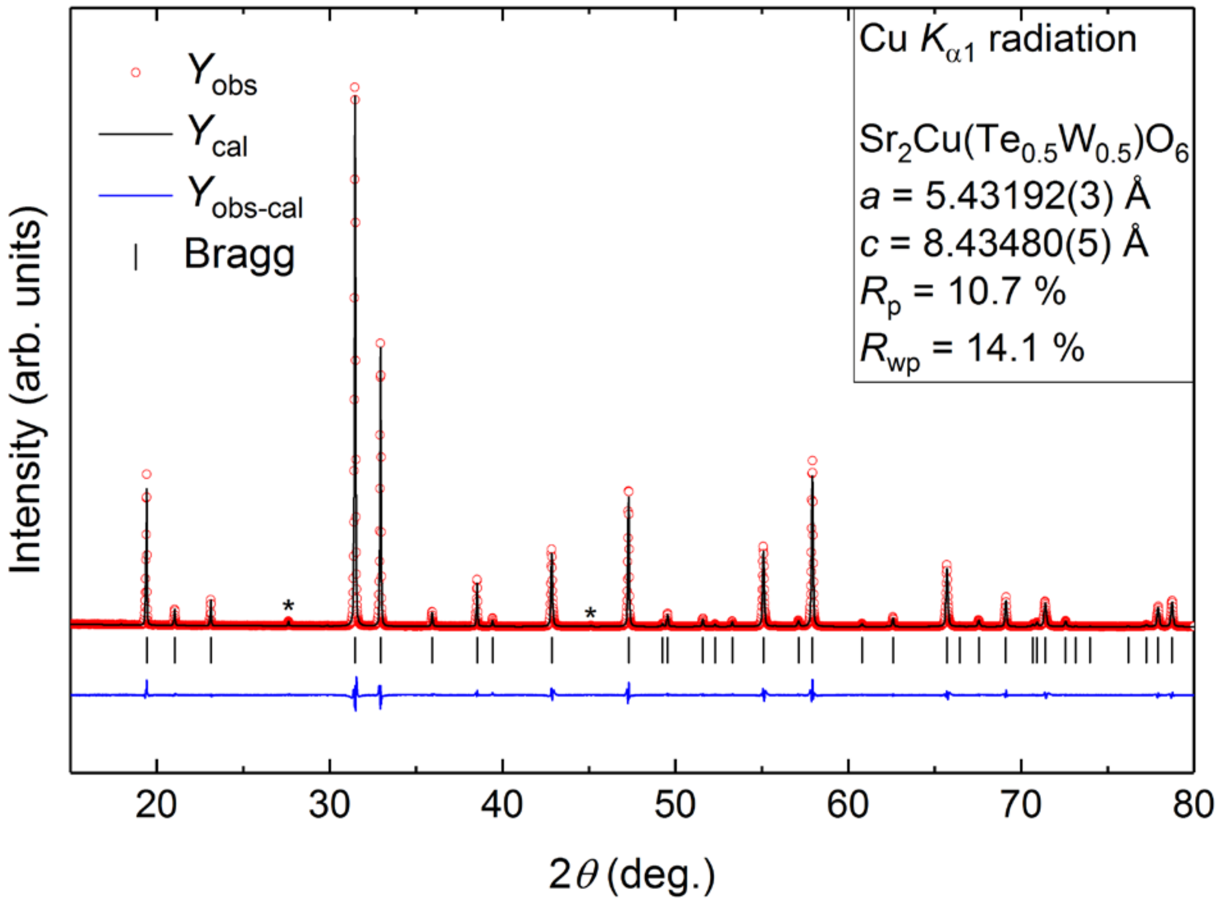


Supplementary Figure 1. Rietveld refinement of $Sr_2Cu(Te_{0.5}W_{0.5})O_6$. Impurity peaks from $SrWO_4$ are marked with an asterisk.

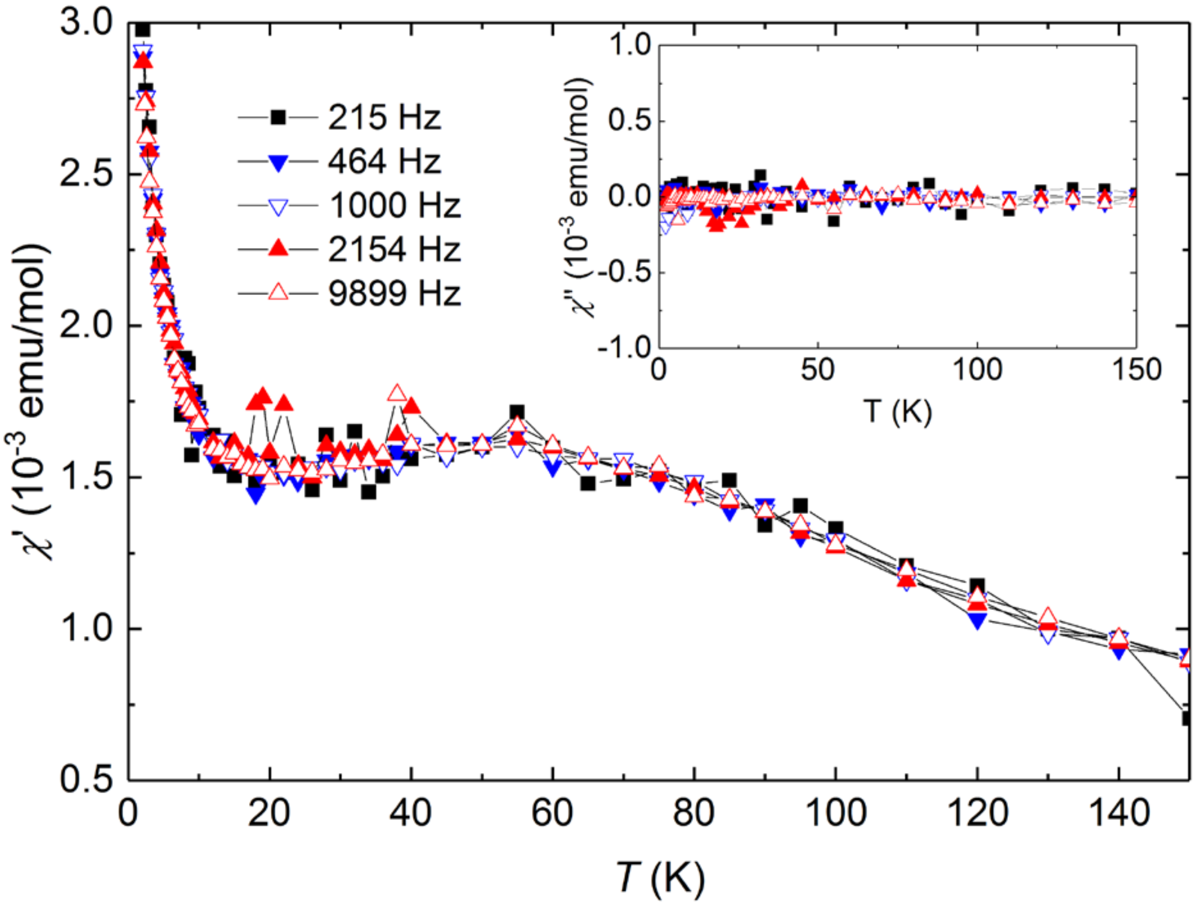


Supplementary Figure 2. The real part of the AC magnetic susceptibility of $Sr_2Cu(Te_{0.5}W_{0.5})O_6$. Inset: The imaginary part of the AC magnetic susceptibility.

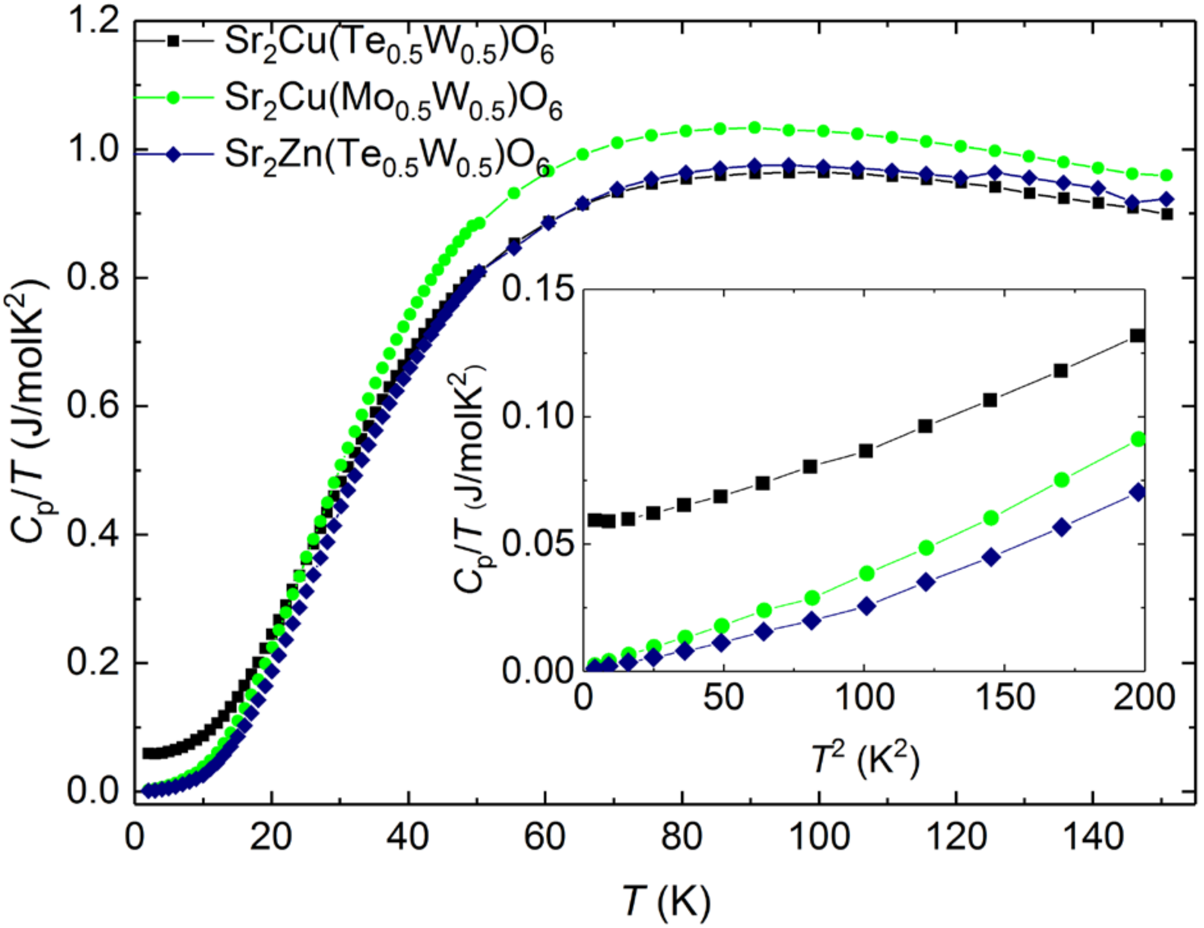


Supplementary Figure 3. Specific heat capacities of $Sr_2Cu(Te_{0.5}W_{0.5})O_6$, $Sr_2Zn(Te_{0.5}W_{0.5})O_6$ and $Sr_2Cu(Mo_{0.5}W_{0.5})O_6$. Inset: Low-temperature $C_p/T$ vs $T^2$ plot.

Supplementary Table 1. Refined crystal structures of $Sr_2Cu(Te_{0.5}W_{0.5})O_6$, $Sr_2CuTeO_6$ and $Sr_2CuWO_6$. Space group *I*4/*m*. Atomic positions W/Te (0, 0, 0), Cu (0, 0, 0.5), Sr (0, 0.5, 0.25), $O_1$ (*x*, *y*, 0), $O_2$ (0, 0, *z*).

| | $Sr_2Cu(Te_{0.5}W_{0.5})O_6$ | $Sr_2CuTeO_6$ | $SrCuWO_6$ |
|---|---|---|---|
| *a* (Å) | 5.43192(3) | 5.43193(2) | 5.42927(1) |
| *c* (Å) | 8.43480(5) | 8.46750(3) | 8.41682(2) |
| $O_1$ *x* | 0.2025(8) | 0.1932(7) | 0.2046(6) |
| $O_1$ *y* | 0.2866(8) | 0.2864(7) | 0.2845(5) |
| $O_2$ *z* | 0.2265(6) | 0.2223(6) | 0.2259(4) |
| $SrWO_4$ (%) | 0.6 | 0 | 0.5 |
| $R_p$ (%) | 10.7 | 11.4 | 7.2 |
| $R_{wp}$ (%) | 14.1 | 14.0 | 9.8 |
